# Metformin-Induced Eosinophilic Interstitial Lung Disease

**DOI:** 10.7759/cureus.38339

**Published:** 2023-04-30

**Authors:** Sami M Alyami, Sarah K Alrasheed, Bashayer A Albogami

**Affiliations:** 1 Pulmonary Medicine, King Abdulaziz Medical City, Riyadh, SAU

**Keywords:** metformin, diabetes type ii, nonspecific interstitial lung disease (nsip), eosinophilic infiltrates, drug induced interstitial lung diseases

## Abstract

Metformin, a mainstay treatment for type two diabetes mellitus has several side effects, ranging from mild to life-threatening ones. One report has found the combination of metformin/glibenclamide a culprit for interstitial lung disease. Other studies have shown that metformin has a protective effect on the lungs. We report a rare case of a 64-year-old male who presented with progressive dyspnea while he was on metformin alone. He was diagnosed with eosinophilic interstitial lung disease (ILD). This was confirmed by a pulmonary function test (PFT), high-resolution chest computed tomography scan (HRCT), and bronchoscopy with bronchoalveolar lavage (BAL). Known causes for eosinophilic pneumonia were excluded, and the patient's condition improved significantly after withdrawing metformin. We report this case due to the rarity of the condition. In fact, this is the only case in the literature, of metformin as the sole agent causing eosinophilic pneumonitis.

## Introduction

Metformin, a biguanide class of antidiabetic medication, has been in use for more than 60 years. Since that time, it continued to be a mainstay treatment for type two diabetes mellitus. Its main mechanism of action is through inhibiting gluconeogenesis in the liver. It has been shown to act via both AMP-activated protein kinase (AMPK)-dependent and AMPK-independent mechanisms, by the inhibition of mitochondrial respiration but also perhaps by the inhibition of mitochondrial glycerophosphate dehydrogenase [1]. It has been reported that one-third of the patients who received metformin have experienced at least one side effect [2]. The side effects of metformin range from gastric upsets and diarrhea to lactic acidosis [3,4]. Numerous drugs have been identified as causes of interstitial lung disease (ILD); the list is growing as new agents are released and establishing a diagnosis of such a disease can be challenging. A case report has found the combination of metformin/glibenclamide to be a culprit for interstitial lung disease [5]. Interestingly, other studies have shown that metformin has a protective effect on the lungs especially from radiation-induced pneumonitis [6]. It has also shown a protective effect in amiodarone-induced interstitial lung disease [7]. We report here a rare case of metformin (as a sole agent) causing eosinophil interstitial lung disease (after excluding other known causes for eosinophilic lung diseases), which was successfully treated by a brief course of steroids and the withdrawal of metformin.

## Case presentation

A 64-year-old former smoker male patient, who quit 20 years ago after a 15-pack-year smoking history, with a medical history significant for type II diabetes mellitus, was referred to our ILD clinic after having an abnormal chest radiograph. He complained of exertional breathlessness, with a Medical Research Council (MRC) severity scale of 3/5 for four months. It was slowly progressive in nature and was associated with dry cough and nonspecific chest discomfort. He also reported a history of generalized fatigability. He denied any cardiovascular red flags like exertional chest, neck, or shoulder pain, palpitations, or intermittent claudication. He also denied infectious symptoms like fever and sputum production. No other respiratory symptoms like wheezing or bluish discoloration were noted. A review of other systems revealed no nausea or vomiting, skin rashes, eye pain, visual changes, joint pains, swellings of joints or glands, hair loss, night sweats, or weight loss. Moreover, there was no history of headaches or any neurological symptoms. There was no family history of connective tissue diseases, parenchymal lung disease, or malignancy. There was also no personal history of alcohol ingestion or illicit drug use. The patient is a retired worker who worked for an oil company with mainly office-based duties and no significant exposures of interest. The patient denied any previous history of contact with animals or any history of recent travel. Further exposure to environmental agents, as well as allergies that may cause respiratory diseases, were excluded after a careful interview. His only medication was metformin, and he denied taking any over-the-counter medication or herbal products. His diabetes was diagnosed six months ago based on high glycosylated hemoglobin levels and he was prescribed metformin 500 mg orally twice daily, which he was compliant on.

The physical exam on presentation revealed a conscious, oriented, middle-aged gentleman who was sitting comfortably on the chair, not in pain or distress. His body temperature was 36.3°C, blood pressure was 163/89 mmHg, heart rate was 98 beats/minute, respiratory rate was 18 breaths/minute, and pulse oximetry was 94% on room air while sitting. The head, eyes, ears, nose, and throat (HEENT) exam was normal with no cyanosis or lymph node enlargement. Chest examination revealed equal breath sounds bilaterally, with few bibasilar end-inspiratory fine crackles. Cardiovascular system examination revealed normal first and second heart sounds with no murmurs, normal jugular venous pressure (JVP), no carotid bruit, and no pedal edema. His abdominal examination was normal with no tenderness, rebound tenderness, guarding, distention, or organomegaly. The musculoskeletal examination was also normal with no joint pain, deformities, or decreased range of motion. A general examination of the skin revealed no skin changes, rashes, or clubbing.

Investigations

The initial evaluation with blood tests revealed a white blood cell (WBC) of 7.10 thousand per cubic milliliter (K/uL) (normal), hemoglobin of 154 grams per deciliter (g/dL) (normal), a platelet count of 203 K/uL (normal), a lymphocytic count of 2.12 (normal), a monocyte count of 0.60 (normal), a basophil count of 0.08 (normal range 0- 0.1×10^9/L), an eosinophil count of 0.22 (normal), and a neutrophil count of 4.29 (normal). Other laboratory investigations revealed a creatinine of 68 umol/L (normal), a blood urea nitrogen (BUN) of 4.6 (normal), potassium of 3.8 (normal), and a bicarbonate level of 21 (normal). Furthermore, his erythrocytes sedimentation rate (ESR) level was 20 millimeters per hour (mm/H) (normal range 0-15 mm/hr) and C-reactive protein was normal. His total immunoglobulin E (IgE) level was 415 KU/L. Other laboratory work-up included a liver profile, lactic acid level, and coagulation profile, which were all within normal limits. Rheumatological workup included antinuclear antibodies (ANA), anti-neutrophil cytoplasmic antibodies (ANCAs), perinuclear anti-neutrophil cytoplasmic antibodies (P-ANCA), anti-cyclic citrullinated peptide (anti-CCP IgG) antibodies, rheumatoid factor, anti-Scl-70 (anti-topoisomerase I) antibodies, anti-La antibodies (anti-SS-B) antibodies, and anti-Ro antibodies (anti-SS-a) antibodies, which were all within the normal range.

Initial pulmonary function testing (PFT) was performed and showed the forced expiratory volume in one second/forced vital capacity (FEV1/FVC) ratio to be 88% (normal range 75%-120%), total lung capacity (TLC) percentage was 56% (normal range > 60%), and diffusion lung capacity for carbon monoxide (DLCO) was 58% (normal 80%-120%). The six minutes walking test showed a significant oxygen drop after walking 355 meters from 93% down to 80%.

**Figure 1:**
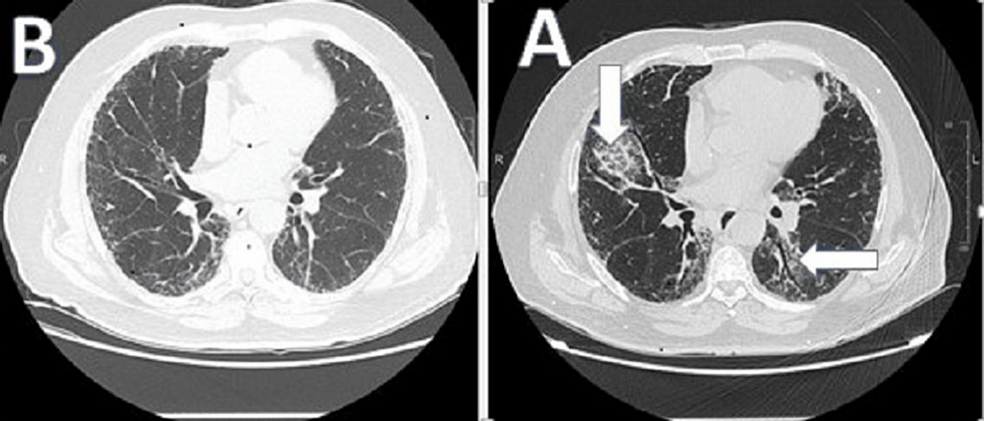
CT of the chest shows the changes before the intervention (A) and the same cut of chest CT scan six months after intervention (B)

**Figure 2:**
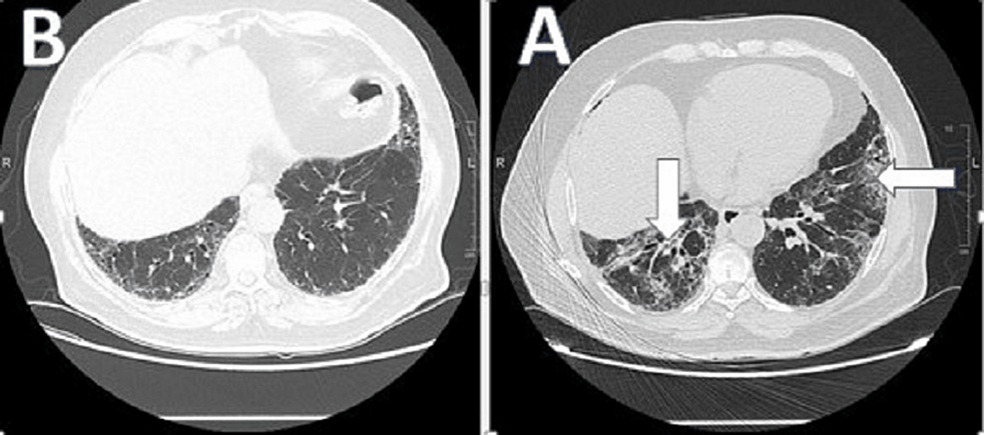
CT of the chest shows the changes before the intervention (A) and the same cut of chest CT scan six months after intervention (B)

A high-resolution chest CT scan (HRCT) showed bilateral predominantly peripheral and basilar reticulations, overlying ground-glass opacities with traction bronchiectasis, air trapping, and few calcified granulomas. The overall HRCT appearance is mostly representing fibrotic nonspecific interstitial pneumonia (NSIP) as per the report of the chest radiologist (Figures 1, 2).

Further investigation by bronchoscopy revealed normal vocal cords and respiratory tract anatomy with no endobronchial lesions. The bronchoalveolar lavage (BAL) taken from both lungs from the lower lobes revealed a high eosinophil percentage of 19% (normal range < 1%) from the right lung and an eosinophil percentage of 51% (normal range <1%) from the left lung BAL. The lymphocytic cell percentage was 6% (normal range 10%-15%) on the right side and 15% in the left lung; the right lower lobe monocyte percentage was 22% (normal range) and the left lower lobe monocyte percentage was 9% (normal range); and right lower lobe segments were 44% (high) and left lower lobe segments were 34% (high). The pathology of BAL was reported as negative for fungal elements, pneumocystis, acid-fast bacilli, and malignant cells.

The above findings were in favor of eosinophilic pneumonitis/ILD. So, further testing to exclude secondary causes for lung eosinophilia (including stool for ova and parasites) was done and all were negative.

Treatment

The only culprit we were left with (before calling it idiopathic) was the medication metformin he was taking for the last six months. Even though we could not find a single case report on metformin (as a sole agent) causing such inflammation, we decided to change metformin to another oral hypoglycemic medication (acarbose) after consultation with an endocrinologist and to give a short course of prednisone 30 mg orally, once daily for two weeks, to accelerate symptoms improvement.

Outcome and follow-up

**Figure 3:**
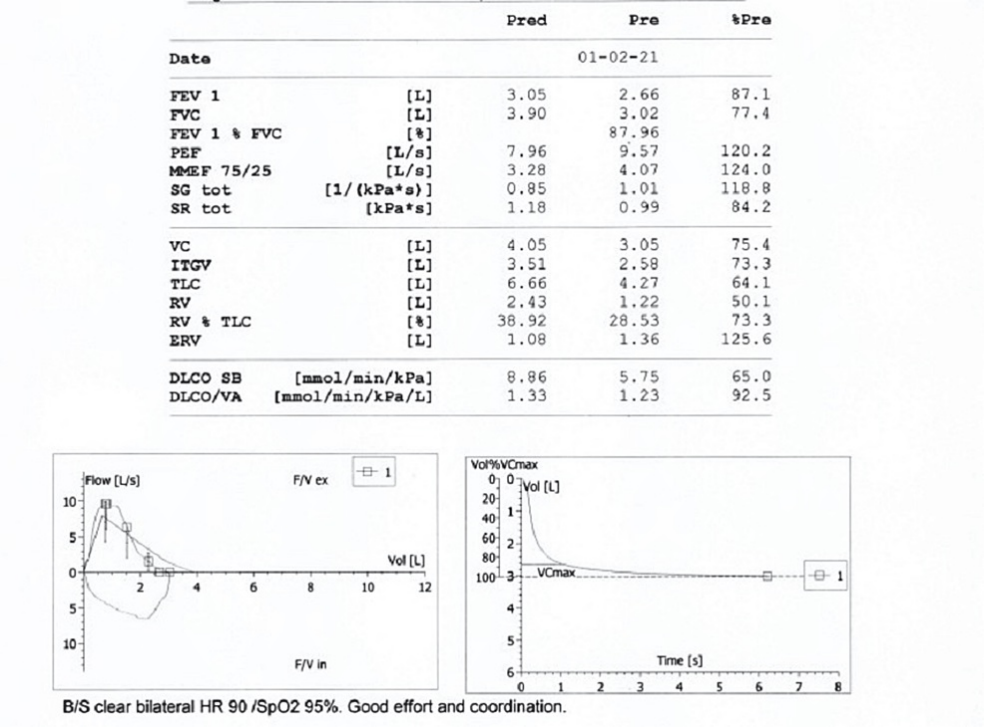
PFT six months after treatment PFT: pulmonary function test; FEV1: forced expiratory volume in 1 second; FVC: forced vital capacity; PEF: peak expiratory flow; MMEF 75/25: mid-expiratory flow; SG tot: specific airways conductance; SR tot: airways resistance; VC: vital capacity; ITGV: intrathoracic gas volume; TLC: total lung capacity; RV: residual volume; DLCO SB: single breath diffusing capacity of the lung; DLCO/VA: diffusion capacity/alveolar volume

The patient was followed clinically in the ILD clinic two weeks later. He reported that his symptoms, including the cough and exertional dyspnea, improved by at least 50%. Further follow-up three months and six months later revealed further clinical improvement in symptoms as per the patient. Repeated PFT (Figure 3) revealed an FEV1/FVC ratio of 88%, a TLC percentage of 56%, a DLCO of 72%, (see attached PFT), and six minutes walk test (Table 1) revealed that he was able to walk 355 meters with much lower oxygen desaturation this time around (95%-90%). Also, a repeated chest CT scan revealed significant improvement in the previously noted NSIP changes (Figures 1, 2).

**Table 1:** Six minutes walking test after intervention

Time (min)	Oxygen saturation %	Heart rate	Distance (meter)
0 (resting)	95	90	355
1	90	98	
2	90	99	
3	91	105	
4	90	102	
5	90	106	
6	91	105	

## Discussion

Drug-induced interstitial lung disease (DILD) is an infiltrative lung disease that results from exposure to a certain drug. Eosinophilic pneumonia characterized by the marked accumulation of eosinophils in lung tissues and/or BAL fluid rarely occurs secondary to drug exposures. Although chronic eosinophilic pneumonia (CEP) responds well to corticosteroids, a relapse occurs frequently. Numerous drugs have been identified as causes of DILD; the list is growing as new agents are released, and establishing the diagnosis of such a disease can be challenging [8,9]. Antidiabetic agents, such as sitagliptin and pioglitazone, have been reported in the literature to be associated with DILD [10]. However, metformin alone was not reported for causing eosinophilic lung disease. On the contrary, other studies have demonstrated the role of metformin as an antifibrotic agent [11]. In this report, we present a rare case of metformin-induced eosinophilic lung infiltrates that mimicked fibrotic ILD, which unexpectedly near completely improved after withdrawing metformin. One might argue that improvement was secondary to prednisone. However, if this was the case, he would not have continued to improve after stopping the short course of prednisone. And if the cause of eosinophilic infiltrates was not metformin induced, the disease would have recurred again over time. Moreover, he continued to get better month after month while avoiding the culprit cause, which was metformin in this case.

## Conclusions

An extensive review of patient medications is a mandatory step when evaluating any new ILD patients. This should include searching for previously reported lung effects for every medication the patient is using. And if possible, the culprit medication/s should be stopped or changed with close follow-up to ensure the causation. Moreover, if the culprit drug has not been reported to cause such adverse effects, one should suspect that this might be the first encounter, similar to what happened in this reported case.
